# Food-Grade Saponin Extract as an Emulsifier and Immunostimulant in Emulsion-Based Subunit Vaccine for Pigs

**DOI:** 10.1155/2018/8979838

**Published:** 2018-11-27

**Authors:** Yulia Burakova, Rachel Madera, Lihua Wang, Sterling Buist, Karen Lleellish, John R. Schlup, Jishu Shi

**Affiliations:** ^1^Department of Anatomy and Physiology, College of Veterinary Science, Kansas State University, 228 Coles Hall, 1620 Denison Ave, Manhattan, KS 66506, USA; ^2^Department of Chemical Engineering, College of Engineering, Kansas State University, 1005 Durland Hall, 1701A Platt St, Manhattan, KS 66506, USA

## Abstract

Subunit vaccines consisting of highly purified antigens require the presence of adjuvants to create effective and long-lasting protective immunity. Advances on adjuvant research include designing combination adjuvants which incorporate two or more adjuvants to enhance vaccine efficacy. Previously, an oil-in-water emulsion adjuvant (OW-14) composed of mineral oil and an inexpensive gum Arabic emulsifier has been reported demonstrating enhanced and robust immune responses when used as an adjuvant in swine subunit vaccines. This study presents a modified version of OW-14 prepared with food-grade *Quillaja* saponin extract (OWq). In new OWq emulsion, saponin extract served as an emulsifier for stabilization of emulsion droplets and as an immunoactive compound. The use of saponins allowed to reduce the required amount of emulsifier in the original OW-14. However, emulsion stabilized with saponins demonstrated extended physical stability even at elevated temperature (37°C). The two-dose vaccination with a classical swine fever virus (CSFV) glycoprotein E2-based vaccine formulated with OWq produced higher levels of E2-specific IgG and virus neutralizing antibodies in pigs in contrast with animals that received the vaccine adjuvanted with oil only. In addition, new OWq adjuvant was safe to use in the vaccination of pigs.

## 1. Introduction

The effectiveness of subunit vaccines relies on immunostimulatory adjuvants to induce potent and long-lasting antigen-specific immune responses. Early adjuvants, like aluminum salts and emulsions, are still the primary choice in vaccine formulations for livestock species because of their safety, simple formulation, and low cost [1, 2]. However, the efficacy of these conventional adjuvants in the induction of antibody responses warrants further improvements. Combination of aluminum salts or emulsions with immunostimulant substances is currently considered to be a promising approach in boosting of the vaccine performance [3, 4]. Combination of the adjuvants with different modes of action presents potential on both enhanced and tailored immune responses for long-lasting protection against the disease. Coadministration of two or more adjuvants in one vaccine has also been explored in veterinary medicine [2]. The combination of different immunostimulant substances, such as saponins, with emulsions and aluminum salts in vaccines for livestock has been actively studied by several research groups [5–7]. For instance, the addition of saponin extract Quil-A® to commercial emulsion-based vaccine was reported to improve humoral immune responses in pigs vaccinated for foot-and-mouth disease [7]. In this present study, we utilized saponin extract, not only as immunostimulatory compound but also as emulsifier to stabilize the emulsion adjuvant.

**Figure 1:**
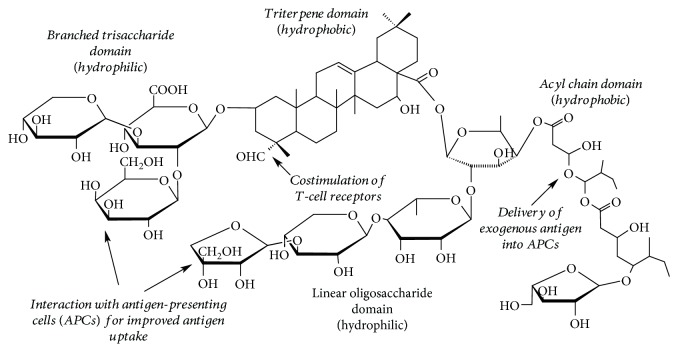
General structure of the saponin molecule reveals the presence of hydrophobic and hydrophilic regions responsible for surface activity of the molecule, while carbohydrate regions, acyl domain, and aldehyde group on triterpene domain demonstrate adjuvant properties. The structure representation is adapted from Yang et al. [14].

Saponins are naturally occurring triterpene glucoside compounds (Figure 1) that are commonly used in vaccine research studies against both animal and human pathogens [8]. *Quillaja saponaria* Molina tree is a main source of saponins for vaccine adjuvants, although there are reports of immunostimulant activity of saponins obtained from other plants [9] and semisynthetic saponins [10]. Commercial saponin adjuvants isolated from *Q. saponaria* tree such as Quil-A (Brenntag Biosector A/S, Denmark) and QS-21 (Desert King, USA) are very potent in inducing high antigen-specific immune responses of both T-helper 1 (Th1) and T-helper 2 (Th2) origins [11]. Studies show that the carbohydrate groups of the saponin molecule interact with receptors on antigen-presenting cells (APCs) and promote antigen phagocytosis and secretion of cytokines by APCs while acyl chain domain assists in the delivery of exogenous antigens into APCs and facilitates Th1 immunity [12]. Aldehyde group on the triterpene domain promotes costimulation of T cell surface receptors (Figure 1) [13]. However, the commercial application of these saponin extracts in animal vaccines is hindered by their high costs.

On the other hand, food-grade *Quillaja* saponin extracts are also used as emulsifiers to produce flavored and vitamin beverages. Saponin molecules contain both hydrophobic and hydrophilic domains making them good candidates for stabilization of oil-in-water food emulsions (Figure 1) [14, 15].

We have previously reported that OW-14, a low-cost emulsion adjuvant based on light mineral oil and food-grade gum Arabic emulsifier, can stimulate high-level antigen-specific antibody responses in vaccines for swine influenza (SI), *Mycoplasma hyopneumoniae*, and classical swine fever (CSF) [16, 17]. In the present study, a variation of the OW-14 emulsion adjuvant formulated with the food-grade saponin extract, Sapnov LS™ (Naturex, USA), in the subunit vaccine for CSF were tested.

CSF is caused by CSF virus, which is a small, enveloped RNA virus in the genus *Pestivirus* of the family *Flaviviridae* [18]. The disease is responsible for economic losses in the swine industry in many countries. Subunit vaccines based on envelope glycoprotein E2 of classical swine fever virus (CSFV) have been shown to induce high-level antigen-specific immune responses and clinical protection of pigs from CSFV challenge [17, 19].

The safety and efficacy of food-grade saponin extract as the emulsifier and immunostimulant in emulsion-based adjuvant coadministered with E2 antigen were investigated. Sapnov LS™ is a water extract of *Q. saponaria* with saponin content around 65% on the dry basis according to the manufacturer. This cost-effective nonionic surfactant is used for production of flavored and colored emulsions such as beverage concentrates and alcoholic drinks. To the best of our knowledge, this is the first report on application of food-grade saponin extract in emulsion-based vaccines for livestock. In addition, an experimental oil-based adjuvant (OBA) that produces an emulsion after low-energy mixing with aqueous solution of E2 antigen was tested in comparison with a saponin-based emulsion vaccine.

## 2. Materials and Methods

### 2.1. Materials

Light mineral oils, Drakeol 5 and Drakeol 6, were purchased from Calumet Panreco (Karns City, PA, USA). TICAmulsion A-2010 emulsifier (gum Arabic) was obtained from TIC Gums (White Marsh, MD, USA). *Quillaja* water extract Sapnov™ (65% saponin content) was provided by Naturex Inc. (Chicago, IL, USA). Polymeric surfactants Atlas G-5002L and Atlox 4916 were obtained from Croda Inc. (New Castle, DE, USA). Medium chain triglyceride oil (MCT oil) was purchased from Jedwards International Inc. (Braintree, MA, USA).

### 2.2. Formulation of Adjuvants and Vaccines

Emulsion adjuvant with saponin extract (OWq) was prepared by dissolving TICAmulsion A-2010 (5% *w*/*v*) in nanopure water and stirred overnight with a magnetic bar. *Quillaja* extract Sapnov emulsifier (0.5% *v*/*v*) was added to the TICAmulsion solution and inverted several times to ensure complete mixing of both emulsifiers in water. Mineral oil Drakeol 5 (15% *v*/*v*) was added to the solution with emulsifiers. The coarse emulsion was mixed with high shear lab mixer (L5MA, Silverson Inc., East Longmeadow, MA, USA) at 10,000 rpm for 15 min and then passed through a microfluidizer M110P (Microfluidics, Westwood, MA, USA) for five times at 10,000 psi. OWq was stored at 4°C until use.

Insect cell-expressed CSFV E2 protein was prepared as described previously [17]. Subunit vaccine was formulated by mixing of 2 vol. of E2 protein solution dissolved in phosphate-buffered saline (PBS) and 1 vol. of OWq emulsion adjuvant using a vortex mixer for several seconds.

Oil-based adjuvant (OBA) was prepared by dissolving two nonionic block copolymer surfactants, Atlas G-5002L and Atlox 4916, in a 7 : 1 by weight ratio mixture of MCT oil and Drakeol 6 mineral oil, respectively. Oils and surfactants were mixed together in an 80 : 20 by weight ratio to produce a clear yellow mixture. To prepare emulsion-based subunit vaccine, OBA was slowly added to the solution of E2 protein in PBS in a 1 : 1 ratio of the adjuvant to aqueous phase and stirred using a magnetic stirrer for 30 min at room temperature.

The final concentration of the antigenic E2 protein in all vaccine formulations was 50 *μ*g per dose.

### 2.3. Physical Characteristics and Stability Study of Adjuvants

Freshly prepared emulsions were analyzed using dynamic light scattering (DLS) with a Malvern Zetasizer Nano ZS 90 (Malvern Instruments, Westborough, MA, USA) to determine droplet size, polydispersity, and zeta potential. The OWq emulsion adjuvant was analyzed as prepared. OBA was mixed with PBS in a 1 : 1 weight ratio using a magnetic stirrer to obtain emulsion for DLS analysis and storage stability assessment.

Approximately 5 *μ*l of each emulsion sample was diluted in 1 ml of deionized water to achieve a translucent solution and perform the measurements. All samples were measured 3 times at room temperature. Results are presented as a mean of 3 measurements ± the standard deviation (SD). The pH values of the emulsions were measured using a VWR SympHony digital pH meter SB21 (VWR International, Radnor, PA, USA), calibrated according to the manufacturer's manual. To assess the shelf stability, samples were stored at 4°C, room temperature (RT), and 37°C and then analyzed again for droplet size and pH after 6 months of storage. Emulsions were also observed visually for the presence of creaming, phase separation, and color change.

### 2.4. Pig Immunization

The animal study was conducted in accordance with Institutional Animal Care and Use Committee (IACUC). Animals were housed at the Large Animal Research Center (LARC) facility, Kansas State University. Conventional Large White-Duroc crossbreed weaned specific-pathogen free piglets (3 weeks of age) were randomly divided into two vaccine groups (*n* = 6 for each group) and 1 negative control group (*n* = 2). Pigs were immunized intramuscularly with 2 ml each of subunit vaccines. The first group received E2 protein co-administered with OWq (E2 + OWq). The second vaccine group was immunized with E2 and oil-based adjuvant (E2 + OBA). Three out of the 6 animals in each vaccine group received a second dose of the subunit vaccines 14 days after the first immunization (two-dose pigs). The negative control group received 2 ml intramuscularly of PBS. Blood samples were collected on days 0, 7, 14, 21, and 28 of the experiment. Sera was separated from the blood and stored at −20°C until further use in assays. Pig weights were measured weekly. Pig health was monitored daily including vaccine injection site reactions. Animals were humanely euthanized and disposed of at the end of the experiment (day 28).

### 2.5. Antibody Responses

E2-specific IgG, IgG1, and IgG2 antibody titers were determined in pig sera by enzyme-linked immunosorbent assay (ELISA) as described previously [17, 20]. Briefly, the 96-well flat-bottom microtiter plates (Corning®) were coated overnight with 62.5 ng/ml of purified E2 followed by washing with ELISA Wash Buffer (0.05% Tween 20 in PBS) and blocked with ELISA Blocking Buffer (2% fetal bovine serum in PBS) for 1 hour at 37°C. Diluted sera were added to plates and incubated for 1 hour at RT, followed by washing 3 times with ELISA Wash Buffer. Goat anti-swine IgG conjugated with horseradish peroxidase (HRP) (#sc-2914, Santa Cruz Biotechnology, USA, dilution 1/1,000), mouse anti-swine IgG1 (#MCA635GA, Bio-Rad Antibodies, USA, dilution 1/300), or mouse anti-swine IgG2 (#MCA636GA, Bio-Rad Antibodies, USA, dilution 1/300) in ELISA Blocking Buffer was added in wells at 100 *μ*l/well as secondary antibodies and incubated 1 hour at RT followed by washing 3 times with ELISA Wash Buffer. HRP-conjugated goat anti-mouse IgG (H+L) (#115-035-003, Jackson ImmunoResearch, USA, dilution 1/1,000) was added to wells at 100 *μ*l/well in case of IgG1 and IgG2 titer analysis and incubated 1 hour at RT followed by washing 3 times with washing buffer. 3,3,5,5-Tetramethylbenzidine- (TMB-) stabilized chromogen (Novex) was used to develop the ELISA plates following by 2N sulfuric acid to stop the reaction. Relative antibody concentration was determined with an optical spectrophotometer using a SpectraMAX microplate reader at 450 nm and was analyzed with Softmax® Pro 6.4 Software (Molecular Devices, USA).

### 2.6. Anti-CSFV Neutralization Assay of Pig Serum

The anti-CSFV neutralizing antibody levels were measured using indirect fluorescent antibody (IFA) assay in pig serum collected on day 21 after the first dose of subunit vaccines or PBS as described elsewhere [20]. Neutralizing titers in serum samples were calculated as the reciprocal of the highest dilution that caused neutralization of the virus in 50% of the wells.

### 2.7. Statistical Analysis

Data from pig experiments were reported as the mean values ± standard error of measurement (SEM). The differences between treatment groups were analyzed by one-way analysis of variance (ANOVA) using SigmaPlot 11.0 software (Systat Software Inc., USA). Differences were considered statistically significant when *p* < 0.05.

## 3. Results

### 3.1. Designed Adjuvants Preserve Their Physical Characteristics after Prolonged Storage at Different Temperatures

**Figure 2:**
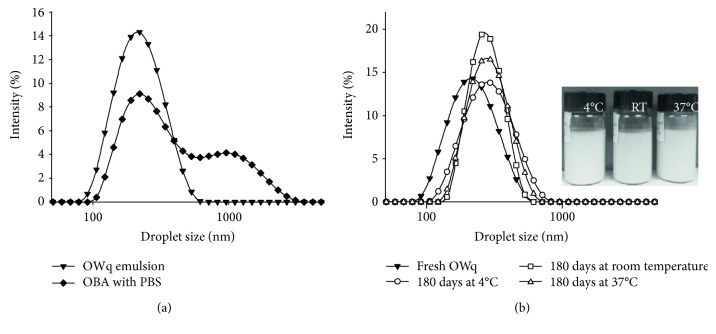
Size distribution of emulsion-based adjuvants obtained with dynamic light scattering (DLS). (a) Freshly prepared OWq emulsion had droplets within 90–600 nm size range, while oil-based adjuvant (OBA) mixed with PBS produced emulsion with droplets between 100 and 3000 nm. (b) After 180-day storage at different temperatures, the slight shift of size distributions towards bigger droplets was detected in OWq emulsion; however, no creaming or phase separation was observed in visual appearance of emulsion samples (photograph insertion).

According to DLS measurements, freshly prepared OWq emulsion adjuvant had nanoscale size of droplets with a mean value of approximately 200 nm and relatively low polydispersity, while emulsion prepared with OBA had droplets around 320 nm with higher difference in droplet sizes (Table 1). All formulations had pH values around 7 (Table 1). The OWq emulsion had a relatively narrow size distribution ranging from 91 to 531 nm, while an emulsion prepared from oil-based adjuvant had droplets with diameters from 106 nm up to 3090 nm (Figure 2(a)). In addition, a saponin-based emulsion had lower zeta potential value (−51.7 mV) than an emulsion prepared with OBA (−25.0 mV) (Table 1). After 6 months of storage at different temperatures, the OWq emulsion adjuvant did not undergo any significant changes in the mean size of emulsion droplets, polydispersity, zeta potential, and pH (Table 1). DLS analysis detected a small shift in the emulsion size distribution towards larger droplets for all OWq samples (4°C, RT, and 37°C) (Figure 2(b)). Thus, after 6-month storage at 37°C, the size of the OWq emulsion droplets ranged from 106 to 955 nm. However, no creaming, phase separation, and color changes were noticed in the appearance of the OWq emulsion after extended storage even at 37°C (Figure 2(b), photograph insertion). The mean droplet size of an emulsion prepared with OBA did not change after storage at 4°C and 37°C. However, a decrease from 320 nm to 217 nm was detected in samples stored at RT (Table 1). Visual observations did not detect any changes in appearance of all OBA emulsion samples (data not shown).

### 3.2. Subunit Vaccine with Saponin-Based Emulsion Did Not Produce Health Issues in Animals and Induced High Antibody Responses

**Figure 3:**
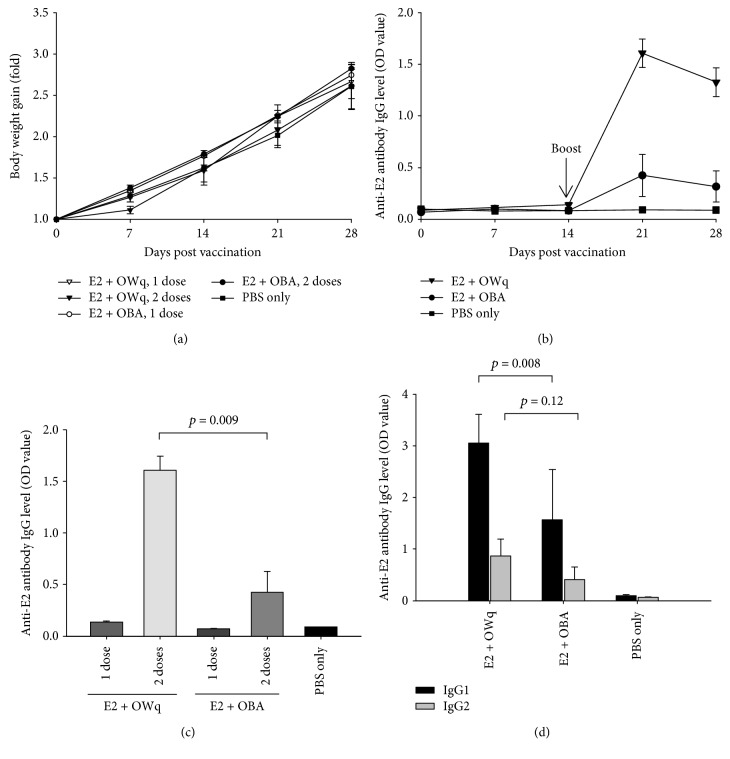
Safety and immunological effect of OWq and OBA injected with E2 antigenic protein. (a) E2 subunit vaccine administered with experimental adjuvants did not impact weight gain in pigs as both one-dose and two-dose animals had grown consistently and at the same level as negative control pigs (PBS only). (b) E2 protein coadministered with OWq adjuvant demonstrated higher E2-specific IgG antibody titer detected on days 21 and 28 in serum samples (dilution 1/5000) of two-dose pigs than subunit vaccine with OBA. (c) Two-dose pigs developed significantly higher IgG titer than animals receiving single vaccination on day 21 of the study (dilution 1/5000). (d) IgG1 and IgG2 titers in serum samples (dilution 1/1000) collected from two-dose pigs on day 21 of the study. Data are presented as a mean ± SEM.

After vaccination with E2 subunit vaccines, all pigs grew consistently, and no difference in weight gain was observed between the animals that received 1 dose and 2 doses of the subunit vaccines and negative control group injected with PBS (Figure 3(a)). The presence of small (1–2 cm) subcutaneous bumps was observed at the injection sites in the E2 + OWq-vaccinated pig groups. By day 28 of study, they decreased in size and mostly disappeared. The negative control group and the E2 + OBA-vaccinated pig group did not have any pathological changes at injection sites (data not shown). No issues were observed in pig health, and all animals survived by the end of the study.

Pigs that received the boost vaccine on day 14 of study had increased E2-specific IgG levels on days 21 and 28 of the experiment (Figure 3(b)). The animals immunized with E2 + OWq had statistically significant higher IgG titers in comparison with the vaccine group that received E2 with OBA (*p* = 0.009) and control group (Figure 3(c)). As expected, a two-dose immunization schedule provided significantly higher IgG titers than one dose in both vaccine groups on day 21 of the study (Figure 3(c)). The IgG1 and IgG2 antibody titers were also higher in E2 + OWq group in comparison with E2 + OBA vaccine group, although it should be noted that there was no statistically significant difference between the two groups (*p* = 0.08 and 0.12 for IgG1 and IgG2 titer analysis, respectively) (Figure 3(d)).

Table 2 represents the results of anti-CSFV neutralization assay performed with pig serum collected on day 21 of the study. The highest neutralizing antibody titers were detected in pigs immunized with two doses of E2 subunit vaccine adjuvanted with OWq emulsion. Very low or no detectable titers were detected in all other pigs including animals inoculated with PBS instead of the subunit vaccine (Table 2).

## 4. Discussion

Emulsions are cost-effective vaccine adjuvants, and their combination with saponins is a promising way to increase the efficacy of veterinary vaccines. Saponins are very effective immunostimulants and have been studied in vaccine formulations for the past decades [2]. However, saponins' high toxicity due to their detergent properties presents a main drawback in their wide application in human and animal vaccines [11]. There are commercially available purified saponin extracts (Quil-A, QS-21); however, their high cost precludes their application as adjuvants in mass livestock immunization.

Safety, efficacy, and physical and chemical stability of the vaccine adjuvants are the key factors in designing novel vaccine formulations. In addition, the cost of materials and production are of paramount importance in the development of livestock vaccines [21]. In this study, a mineral oil-based emulsion with a food-grade inexpensive saponin extract (OWq) and oil-based adjuvant that produces an emulsion after gentle mixing with an aqueous phase were tested for safety and immunological activity in swine vaccination.

In OWq, the saponin extract served as an emulsifier for stabilization of emulsion droplets and as an immunostimulant in the subunit vaccine. Thus, the addition of saponin extract to original formulation (OW-14) [16] allowed reducing the amount of TICAmulsion A-2010 emulsifier from 7.5% *w*/*v* to 5% *w*/*v* without affecting the shelf stability of adjuvant. Moreover, OWq emulsion adjuvant demonstrated good physical stability after 180 days of storage at all tested temperatures. Mean droplet size remained less than 300 nm with low polydispersity among the droplets (Table 1, Figure 2(b)). The slight drop in pH values in samples stored at 4°C and 37°C was detected after 180 days. However, no physical changes in emulsion samples, such as creaming or phase separation, were observed. Physical stability is very important for livestock vaccines, especially in developing countries where refrigeration during transportation and storage of the vaccines is not always available or cost-effective.

The droplet diameters, polydispersity, and zeta potential of prepared emulsions are important factors in predicting the physical stability of the emulsions. Freshly prepared OWq emulsion had nanosize droplets in a range from 91 to 531 nm and low zeta potential (−51.7 mV), which provides electrostatic repulsion between the droplets and prevents their fast flocculation and coalescence. Vaccine can be easily prepared at the site by simple hand mixing or gentle agitation of OWq with antigenic solution. It can be readily administered through a standard syringe with 20-gauge needle.

OBA mixed with PBS by a low-energy mixing method produced an emulsion with high polydispersity, mean droplet size around 320 nm, and zeta potential around −25 mV. No significant changes in mean droplet size were detected after 180-day storage. However, a decrease in zeta potential value indicates lower colloidal stability over time in comparison with OWq emulsion.

The safety and immunological activity of designed adjuvants were tested in swine vaccination with E2 antigen. Pigs immunized with experimental adjuvants in subunit CSFV E2 vaccines did not experience any health issues and gained the weight on the same level as negative control pigs during the entire study (Figure 3(a)). Small subcutaneous bumps were observed at the injection sites of the OWq vaccine group, although they decreased in size significantly or disappeared towards the end of the experiment. These findings suggest that food-grade saponin extract can be safely utilized in veterinary vaccine formulations. Moreover, the group immunized with two doses of E2 subunit protein adjuvanted with OWq emulsion had higher E2-specific IgG levels (Figures 3(b)–3(d)) and anti-CSFV neutralizing antibody titers (Table 2) in comparison with vaccine group that received E2 formulated with emulsion and without saponins or other immunostimulant compounds.

The reason E2 + OBA vaccine did not induce strong IgG antibody responses and high anti-CSFV neutralizing titers can be attributed to the composition and physical characteristics of the adjuvant. OBA predominantly consisted of plant-derived MCT oil with small portion of mineral oil, while vaccines based on mineral oil generally produce more irritation at the injection sites and induce higher immune responses than vaccines with plant-derived oils [22]. In addition, the mean droplet size of emulsion fabricated from OBA was 327.40 nm. However, it has been demonstrated that particles less than 200 nm are processed more efficiently by the APCs [23]. Clearly, the changes in composition of the OBA should be done to reduce the size of emulsion droplets and improve the emulsion stability and efficacy as a vaccine adjuvant.

Animals that were immunized twice with subunit vaccines have developed significantly higher IgG titers (Figure 3(c)) and anti-CSFV neutralizing antibody titers (Table 2) in comparison with pigs receiving single vaccination. However, previous studies demonstrated that even one-dose vaccination with an emulsion-based subunit vaccine can protect the pigs from the challenge with CSF virus [17, 20].

Previous findings have shown that saponins promote the production of IgG2 over IgG1 antibody subclass and favor Th1 and cytotoxic T lymphocyte responses in contrast with conventional adjuvants such as aluminum salts and emulsions in murine models [9, 11]. These suggest that saponin adjuvants can be beneficial in vaccines against intracellular pathogens such as viruses. In the present study, IgG2 titer analysis of pig serum samples did not show statistically significant differences between saponin-based emulsion adjuvant and an emulsion without saponins (*p* = 0.12) (Figure 3(d)). This can be attributed to several factors such as the difference between mice and swine immune responses to the saponins, heterogeneous composition of food-grade saponin extract, or poor stability of the saponins resulting in stimulation of Th2 immunity rather than Th1. It has been demonstrated that various fractions of *Q. saponaria* extract have different immunological activity and can induce the production of different IgG antibody subclasses [11]. Another study showed that *Q. saponaria* extract are easily degraded during storage resulting in deacylation of the saponin molecules and the inability to promote a strong Th1 response and IgG2 production [24]. The exact fraction composition of the food-grade saponin extract used in this study is unknown and needed to be determined to verify these speculations. In addition, further investigation is required to confirm the level of protection of swine immunized with E2 protein and OWq saponin-based emulsion after the challenge with CSF virus.

## 5. Conclusion

Inexpensive food-grade saponin extract was employed to create emulsion-based adjuvant for swine subunit vaccine. Saponins served a dual purpose: stabilization of emulsion droplets and stimulation of immune responses. The addition of saponin extract helped to reduce the amount of emulsifier in the original OW-14 adjuvant. This change in composition did not impact droplet size stability of the emulsion adjuvant. Thus, OWq can be stored at least 180 days at different temperatures without dramatic changes in droplet size, creaming, or phase separation. A saponin-based emulsion adjuvant was safe to use in swine immunization as no significant inflammations at the injection sites and consistent weight gain in animals were observed after coadministration with E2 antigenic protein. Two doses of CSF E2 subunit vaccine with OWq adjuvant demonstrated high total IgG and anti-CSFV neutralizing antibody titers in pigs in comparison with the emulsion vaccine prepared with oil-based adjuvant without saponins. This is the first demonstration that a cost-effective food-grade saponin extract can be incorporated in an emulsion-based adjuvant and safely used in livestock immunization promoting strong antibody responses.

## Figures and Tables

**Table 1:** Physical characteristics of fresh emulsion adjuvants and after 180-day storage at different temperatures.

Emulsion		Mean droplet size ± S.D., nm	Polydispersity ± S.D.	Zeta potential ± S.D., mV	pH
OWq	Fresh	203.40 ± 1.83	0.115 ± 0.008	−51.7 ± 0.2	7.1
	180 days at 4°C	272.50 ± 4.86	0.117 ± 0.025	−53.2 ± 0.7	6.2
	180 days at RT	266.57 ± 5.73	0.054 ± 0.035	−58.0 ± 3.1	7.1
	180 days at 37°C	277.80 ± 4.98	0.142 ± 0.022	−56.0 ± 3.9	6.3

OBA in PBS	Fresh	327.40 ± 4.59	0.270 ± 0.004	−25.0 ± 3.7	7.3
	180 days at 4°C	310.37 ± 2.29	0.193 ± 0.005	−12.4 ± 0.2	7.2
	180 days at RT	217.97 ± 1.85	0.147 ± 0.010	−6.8 ± 0.4	7.2
	180 days at 37°C	367.37 ± 0.51	0.242 ± 0.012	−11.5 ± 0.2	7.2

RT: room temperature; SD: standard deviation.

**Table 2:** Anti-CSFV neutralizing antibody titers (ND_50_) in pigs on day 21 of the study.

Treatment group	Pig #	Day 21
E2 + OWq, 1 dose	178	<5
	181	5
	185	5

E2 + OWq, 2 doses	186	640
	188	960
	195	480

E2 + OBA, 1 dose	177	<5
	180	<5
	187	<5

E2 + OBA, 2 doses	189	480
	190	20
	193	40

PBS only	184	<5
	191	<5

## Data Availability

The datasets used to support the findings of this study are available from the corresponding author upon reasonable request.
